# Characterization of a novel β-barrel protein (AtOM47) from the mitochondrial outer membrane of *Arabidopsis thaliana*

**DOI:** 10.1093/jxb/erw366

**Published:** 2016-10-06

**Authors:** Lu Li, Szymon Kubiszewski-Jakubiak, Jordan Radomiljac, Yan Wang, Simon R. Law, Olivier Keech, Reena Narsai, Oliver Berkowitz, Owen Duncan, Monika W. Murcha, James Whelan

**Affiliations:** ^1^Australian Research Council Centre of Excellence in Plant Energy Biology, Department of Animal, Plant and Soil Science, School of Life Science, La Trobe University, Bundoora, Victoria, 3086, Australia; ^2^Australian Research Council Centre of Excellence in Plant Energy Biology, University of Western Australia, 35 Stirling Highway, Crawley, Western Australia, 6009 Australia; ^3^Department of Plant Physiology, Umeå Plant Science Centre, Umeå University, S-90187 Umeå, Sweden

**Keywords:** Arabidopsis, At3g27930, β-barrel protein, membrane transport, mitochondria, OM47, senescence, VDAC.

## Abstract

In plant cells, mitochondria are major providers of energy and building blocks for growth and development as well as abiotic and biotic stress responses. They are encircled by two lipid membranes containing proteins that control mitochondrial function through the import of macromolecules and metabolites. Characterization of a novel β-barrel protein, OUTER MEMBRANE PROTEIN 47 (OM47), unique to the green lineage and related to the voltage-dependent anion channel (VDAC) protein family, showed that OM47 can complement a VDAC mutant in yeast. Mutation of *OM47* in *Arabidopsis thaliana* by T-DNA insertion had no effect on the import of proteins, such as the β-barrel proteins translocase of the outer membrane 40 (TOM40) or sorting and assembly machinery 50 (SAM50), into mitochondria. Molecular and physiological analyses revealed a delay in chlorophyll breakdown, higher levels of starch, and a delay in the induction of senescence marker genes in the mutant lines. While there was a reduction of >90% in OM47 protein in mitochondria isolated from 3-week-old *om47* mutants, in mitochondria isolated from 8-week-old plants OM47 levels were similar to that of the wild type. This recovery was achieved by an up-regulation of *OM47* transcript abundance in the mutants. Combined, these results highlight a role in leaf senescence for this plant-specific β-barrel protein, probably mediating the recovery and recycling of chloroplast breakdown products by transporting metabolic intermediates into and out of mitochondria.

## Highlight

Characterization of a novel β-barrel protein in Arabidopsis identifies its role in leaf senescence by mediating the transport of chloroplast breakdown products across the outer mitochondrial membrane for their recycling.

## Introduction

Eukaryotic cells are characterized by the presence of membrane-bound compartments, known as organelles, which carry out various functions and contain a specific subset of the cellular proteome. Mitochondria and plastids are endosymbiotic organelles in that their origin can be attributed to a symbiotic event occurring ~2 billion and 1 billion to 500 million years ago, respectively (Gould *et al.*, 2008; Lane and Martin, 2010). All organelles require the transport of various metabolites and compounds across the membrane(s), and thus transmembrane transport proteins are intensively studied in cell biology. Mitochondria and plastids are somewhat different from other organelles in that they contain two or more membrane structures. Both mitochondria and plastids possess outer and inner membranes; chloroplasts also harbour thylakoid membranes within their stroma. While the diverse array of proteins involved in primary metabolism and electron transport in plastids (more specifically chloroplasts) and mitochondria have been identified and intensively studied, the protein components of the outer membrane of both these organelles remain less well understood. For both mitochondria and chloroplasts, the outer membrane defines the organelle’s boundary and thus its interaction with the rest of the cell. These membranes are considered semi-permeable because they contain β-barrel-type proteins that facilitate the passage of molecules and metabolites of up to ~5kDa (Homble *et al.*, 2012).

Homologous β-barrel proteins are also present in Gram-negative bacteria. They are comprised of a number of β-sheets, ranging from eight to 24 (Fairman *et al.*, 2011), that form a pore which further allows the passage of molecules through a membrane. Interestingly, ~50 β-barrel proteins are known to be encoded by many bacterial genomes (Wimley, 2003); and it is therefore not surprising that both chloroplasts and mitochondria have retained some of these β-barrel proteins after endosymbiosis occurred. For instance, in *Arabidopsis thaliana*, the few known β-barrel proteins in chloroplasts are: the three outer envelope proteins 21, 24, and 37 (OEP 21, OEP 24, and OEP37) that act as anion or cation transporters; the translocon outer envelope membrane complex 75 (TOC75), of which there are multiple isoforms involved in protein import; and the dimeric β-barrel endoplasmic reticulum to chloroplast lipid transfer protein trigalactosyldiacylglycerol 4 (TGD4) (Simm *et al.*, 2013; Oh and Hwang, 2015). Mitochondria generally contain three types of β-barrel proteins. These include four isoforms of the voltage-dependent anion channel (VDAC), which are generally involved in metabolite exchange (Robert *et al.*, 2012; Tateda *et al.*, 2011); the two isoforms of the translocase of the outer mitochondrial membrane 40 (TOM40), the primary channel by which almost all proteins enter the mitochondrion (Murcha *et al.*, 2015); and the two isoforms of the sorting and assembly machinery 50 (SAM50) protein that is specifically involved in the insertion of β-barrel proteins into the outer membrane (Murcha *et al.*, 2015). These are the three types of β-barrel protein common in most but not all eukaryotes. Exception or additions to this general picture include MDM10 (mitochondrial distribution and morphology protein), which is the most recently characterized mitochondrial β-barrel protein and to date only reported in fungi. It is involved in the assembly of the outer membrane protein complexes and a constituent of the endoplasmic reticulum–mitochondria encounter structure (ERMES) complex, best characterized in yeast (Meisinger *et al.*, 2004; Flinner *et al.*, 2013). Trypanosomids appear to lack a TOM40 orthologue and use an alternative β-barrel protein, called archaic translocase of the outer mitochondrial membrane (Pusnik *et al.*, 2012).

The three types of mitochondrial β-barrel proteins outlined above have been studied in plants to varying degrees. The most extensively studied family is the VDAC family, the most abundant protein in the outer membrane. In Arabidopsis, the four genes encoding VDAC have varied and important functions with respect to plant development such as pollen development (Tateda *et al.*, 2011), regulation of respiration during seed germination at low temperatures (Yang *et al.*, 2011), and in the plant stress response (Homble *et al.*, 2012; Takahashi and Tateda, 2013). There are a limited number of studies with regards to the other plant mitochondrial β-barrel proteins TOM40 and SAM50, both involved in protein transport. While two genes code for TOM40 in Arabidopsis, the major pore-forming translocase responsible for the import of the majority of the mitochondrial proteome, only one is thought to play a major role, as deletion results in an embryo lethal phenotype. SAM50 is responsible for the insertion of β-barrel proteins into the outer membrane (Lister *et al.*, 2007; Duncan *et al.*, 2013).

A biochemical characterization of the mitochondrial outer membrane proteome from Arabidopsis revealed 42 proteins with functions putatively associated with mitochondrial morphology and lipid synthesis, as well as a number of others for which the function remains unknown based on sequence comparisons alone (Duncan *et al.*, 2011). Although this study added 27 novel proteins, thus more than doubling the confirmed number of mitochondrial outer membrane proteins, it is likely that more proteins with related functions are present in the mitochondrial outer membrane. In comparison, there are >100 proteins predicted from a variety of sources of data to be part of the mitochondrial outer membrane in yeast (Zahedi *et al.*, 2006).

Here we report the characterization of a novel plant mitochondrial β-barrel protein, previously identified by biochemical characterization (Duncan *et al.*, 2011). Combining phenotypical, physiological, biochemical, and molecular analyses, we reveal that this 47kDa outer membrane protein, named OM47, plays a role in plant development. The *om47* knockout plants display a delay in leaf senescence, leading to a prolonged life span and enhanced vegetative growth.

## Materials and methods

### Plant material and growth conditions

*Arabidopsis thaliana* Columbia (Col-0) plants were used as the wild type in this study. Homozygous T-DNA insertion lines for *om47-1* (SALK_016767; http://abrc.osu.edu/) and *om47-2* (GABI_369G03; http://www.gabi-kat.de/) were obtained and genotyped by PCR (Fig. 3Ai; Supplementary Table S1 at *JXB* online). The sites of insertions were confirmed by Sanger sequencing of the amplified PCR products (Supplementary Fig. S1).

Phenotypic analysis was carried out according to Boyes *et al.* (2001) on plants grown on either soil or Murashige and Skoog (MS) media. For plate-based phenotyping, seeds were surfaced-sterilized with 70% (v/v) ethanol and germinated on MS medium containing 4.3g l^–1^ (w/v) MS powder, 0.5g l^–1^ MES, and 1ml l^–1^ Gamborg B5 vitamins (pH 5.7) with varying concentrations of sucrose. Plates were stratified for 48h at 4 °C before being transferred to growth conditions. Root length of 50 plants per line per condition was measured at day 6, day 10, and day 14, respectively. Analysis of root length was carried out using Image J. For soil-based phenotyping, all lines were grown in a randomized design on soil mix consisting of grade 2 vermiculite, perlite, and soil in a 1:1:3 ratio. All growth conditions were at 22 °C/22 °C (day/night), 65% relative humidity, 120 μmol m^−2^ s^−1^ photosynthetic photon flux density (PPFD) and 16h/8h (day/night) photoperiod unless specified otherwise. All measurements were carried out in biological triplicate.

### Bioinformatic analysis

Protein domains in the OM47 predicted protein were identified using the Conserved Domains database (NCBI CDD; http://www.ncbi.nlm.nih.gov/Structure/cdd/wrpsb.cgi) (Marchler-Bauer *et al.*, 2013). The β-strands were predicted using the transmembrane strands and topology of the β-barrel outer membrane protein prediction web server (PRED TMBB; http://bioinformatics.biol.uoa.gr/PRED-TMBB/) based on a hidden Markov model (Bagos *et al.*, 2004). The predicted amino acid sequences encoding a variety known of β-barrel proteins were retrieved from the Arabidopsis Information Resource (TAIR; http://www.arabidopsis.org/) TAIR10 genome release. The corresponding yeast sequences were obtained from GenBank using a text search.

Phylogenetic analysis was carried out using MEGA 5.2.2 (Tamura *et al.*, 2011). The full-length protein alignments were first generated using the Clustal option, and the phylogenetic tree was created using the maximum likelihood tree method and the Jones–Thornton–Taylor model after 1000 replications.

### *In silico* developmental expression analysis

Heatmaps illustrating developmental expression analysis were generated *in silico* in the Partek genomics suite (version 6.6) using GC-RMA normalized publicly available microarray data from the AtGenExpress developmental set (Schmid *et al.*, 2005) and the Arabidopsis germination data set, as described previously (Narsai *et al.*, 2011). The heatmap illustrating expression levels for At3g27930 in *A. thaliana* at different stages of plant development were generated using the development tool in GENEVESTIGATOR (ID: 256851_at) and the web-browser data mining interface for Affymetrix GeneChip data (https://genevestigator.com) (Hruz *et al.*, 2008). The transcript abundance patterns of genes encoding β-barrel proteins during senescence was normalized separately using the highest value set to 1 for each gene and data taken from Breeze *et al.* (2011).

### Yeast transformation for functional complementation

Functional complementation of a VDAC protein by OM47 was carried out using ScVDAC1(–), a yeast mutant strain lacking VDAC1 protein (*por*) (YNL055c; MATα, *his3Δ1*; *leu2Δ0*; *lys2Δ0*; *ura3Δ0*; *UNL055c::kanMX4*) (Lee *et al.*, 1998), and a wild-type yeast strain (BY4742; MATα, *his3Δ1*; *leu2Δ0*; *lys2Δ0*; *ura3Δ0*) according to the experimental design previously described (Lee *et al.*, 2009).

The full-length coding sequence of OM47 was cloned into pDONR201 using Gateway technology and recombined into the yeast expression vector pAG426GPD (Alberti *et al.*, 2007) under the constitutive promoter GPD and the URA3 selectable marker, and transformed into YNL055c. As a positive control, YNL055c was also cloned into pAG426GPD and transformed into the yeast VDAC (ScVDAC) deletion strain.

Transformed strains (YNL055c+ScVDAC and YNL055c+OM47) along with BY4742 and untransformed YNL055c were plated on synthetic defined (SD) media (6.7g l^–1^ yeast nitrogen base without amino acids, 0.6g l^–1^ amino acid dropout powder) at 2% (w/v) glucose for 3 d, or 2.5% (v/v) glycerol for 5 d at 30 °C.

### Dark-induced senescence assay

Two types of dark-induced senescence assays were carried out. On attached leaves, 3-week-old Col-0 and *om47* mutants had leaves 5, 6, and 7 covered with aluminium pouches for a number of days, 3 d or 8 d as indicated; with control treated lines grown without aluminium pouches (Weaver and Amasino, 2001). For detached leaf dark-induced senescence assay treatments, 4-week-old Col-0 and *om47* mutants had leaves 5, 6, and 7 excised, covered in aluminium foil, and placed inside foil-wrapped Petri dishes for 3 d, with Whatman 3MM paper moistened with water to prevent drying.

### Gas exchange

Fully expanded leaves were enclosed into a 1cm reach chamber (Li-COR, 6400-15) attached to a Li-COR portable photosynthesis system (BioScientfic Ltd) according to Li *et al.* (2014) with modifications. Gas exchange parameters including net photosynthetic CO_2_ fixation rate, stomatal conductance, and transpiration were measured at 23 °C with a PPFD of 400 μmol m^−2^ s^−1^ (using the Li-COR 6400-18A Red, Green, Blue Light Source) and CO_2_ concentration of 400 μmol mol^−1^. The rate of mitochondrial respiration in the dark was determined by measuring the net photosynthetic CO_2_ fixation rate at 0 μmol m^−2^ s^−1^ PPFD.

### Chlorophyll analysis and fluorescence

Three covered or non-covered leaves per plant and five plants for each genotype were snap-frozen in liquid nitrogen and ground well using a TissueLyser II (QIAGEN). A 50mg aliquot of crushed leaf material was incubated with 1.5ml of 80% pre-chilled acetone for 2min and thoroughly mixed for 5min. Samples were cleared by centrifugation at 14 000rpm for 1min and the supernatant was kept in new pre-chilled tubes. The above procedure was repeated for the remaining samples and all supernatants were mixed together. Chlorophyll was then quantified at 663nm and 646nm with a spectrophotometer (BMG, ClarioSTAR) as previously described (Lichtenthaler and Wellburn, 1983).

Chlorophyll fluorescence in *F*_v_/*F*_m_ (maximum quantum yield of PSII) was determined as previously described (Rossel *et al.*, 2006). After a short dark acclimation, plants were pulsed with 120 µmol m^−2^ s^−1^ of actinic light using the IMAGING-PAM M-series Chlorophyll Fluorescence System (Walz).

### Starch analysis and histochemical staining

Starch was extracted and analysed with the help of a plate reader (BMG, ClarioSTAR) using the NAD(P)H-linked assay (Sanjaya *et al.*, 2011). All samples were analysed in biological triplicate. Changes in the absorbance were determined by performing endpoint assays before and after the addition of each enzyme.

The histochemical staining of leaf starch was carried out as described previously (Zhang *et al.*, 2012). Briefly, leaves were incubated in 75% (v/v) ethanol and 25% (v/v) acetic acid for 48h to remove the chlorophyll, followed by three washes in MilliQ water. Staining was achieved by further incubation in 50% (v/v) Lugol solution (Sigma-Aldrich) for 5min. Plant tissue was then destained for 90min in MilliQ water. Leaves were collected at the start (ZT0) and end (ZT16) of the light period. Attached senescent leaves 5, 6, and 7 or detached dark-induced senescent leaves 5, 6 7, 8, and 9 of each plant were collected, and three plants per genotype in each treatment were sampled.

Reactive oxygen species (ROS) in the form of O_2_^−^ were determined as previously described (Sedigheh *et al.*, 2011). Leaves were stained using a 600 μM solution of nitroblue tetrazolium (NBT) for 3h and incubated for 24h in 80% ethanol, twice, in order to remove the chlorophyll. Five leaves per plant and three plants per genotype were sampled.

### Isolation of mitochondria

For the isolation of mitochondria from water-cultured plants, mutant and Col-0 seeds were surfaced-sterilized with 70% (v/v) ethanol and grown in half-strength MS medium containing 2.15g l^–1^ (w/v) MS powder, 0.53g of Gamborg’s B5 salts, 1% (w/v) sucrose, 50 μg ml^–1^ cefotaxime, and 2mM MES/KOH, pH 5.7, with continuous shaking. Plants were grown for 2 weeks and mitochondria isolated as described previously (Lister *et al.*, 2007). For the isolation of mitochondria from soil-grown plants, mutant and Col-0 seeds were sown directly onto soil and stratified for 48h at 4 °C before being transferred to 16h light/8h dark growth conditions. Plants were grown for 3, 4, 7, and 8 weeks, and mitochondria were isolated as described previously (Murcha and Whelan, 2015).

### Immunodetection

Mitochondrial protein was separated by SDS–PAGE (Bio-Rad, Sydney), transferred to Hybond-C extra nitrocellulose (Bio-Rad, Sydney, Australia), and immunodetected as described previously (Wang *et al.*, 2012) (ST2). To ensure linearity of detection, 2, 4, and 8 µg of mitochondrial proteins were loaded. The intensity of the cross-reacting bands was quantitated using Image Lab™ software (Bio-Rad). The pixel densities were expressed relative to the wild type (Col-0, 2 µg), where the pixel density value was set to 1. Three biological replicates were performed and the averages determined. Significant changes were determined using Student’s *t*-test, with an asterisk indicating a significant difference with a *P*-value ≤0.05. Antibodies used were raised against Ndufs4 (Meyer *et al.*, 2009), NDB2 (Soole and Smith, 2015), AOX (Elthon *et al.*, 1989), Porin (Lister *et al*., 2004), Tim9, Tim50 (Wang *et al.*, 2012), TOM40, RISP (Duncan *et al.*, 2011), SAM50, Erv1 (Carrie *et al.*, 2010), COXII, cytochrome *c*, ATP β-subunit (Agrisera), and KDSB (Duncan *et al.*, 2011). To generate antibodies against OM47 and ELM1 (At5g22350), cDNA of OM47 and ELM1 (amino acids 101–200) was cloned into pDONR201 and then recombined into pDEST17 using Gateway^®^ technology (Invitrogen). The recombinant protein was expressed in *Escherichia coli* strain BL21 (DE3) pLys (Stratagene, La Jolla, CA, USA), and purified by denaturing immobilized metal affinity chromatography (IMAC) using the Profinia protein purification system (Bio-Rad). Purified protein was confirmed by MS prior to inoculation into rabbits according to the standard protocol of Cooper and Paterson (2009).

### RNA isolation and digital quantitative RT–PCR

Total RNA from ~60mg of frozen homogenized *A. thaliana* tissue was isolated using the RNeasy Plant mini kit (Qiagen, Sydney, Australia) according to the manufacturer’s instructions with modifications. The RNA was eluted in molecular grade DNase- and RNase-free water and analysed on agarose gels prior to reverse transcription and PCR. The cDNA was synthesized by reverse transcription of the total RNA using the iScript™ cDNA Synthesis Kit (Bio-Rad, Hercules, CA, USA) according to the manufacturer’s instructions. Subsequently, absolute transcript abundances were assayed using the QX200 Droplet Digital System using EvaGreen ddPCR Supermix (Bio-Rad, Gladesville, NSW, Australia) in biological triplicates. Significant changes were determined using Student’s *t*-test, with an asterisk indicating a significant difference with a *P*-value ≤0.05. Primers used for ddPCR are listed in Supplementary Table S1.

To ensure that the correct leaf was chosen from each plant, leaf number and position were marked after emergence, and tracked on a daily basis to ensure that the correct leaf age was chosen.

## Results

### Bioinformatic analysis reveals OM47 as a Porin3 β-barrel protein

**Fig. 1.**
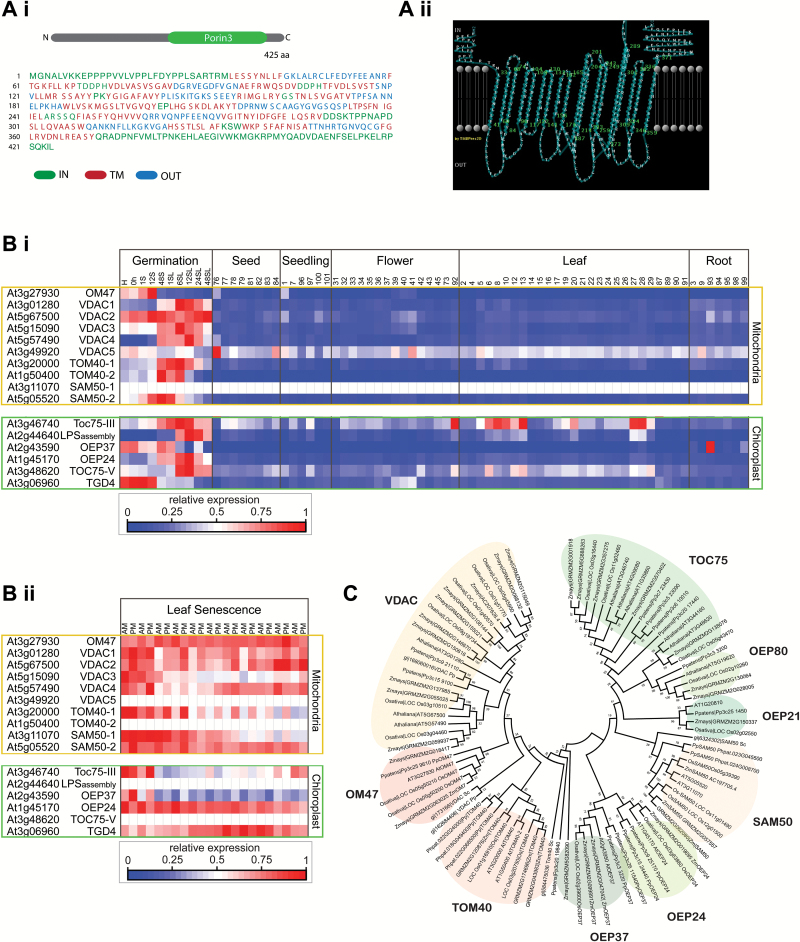
Bioinformatics analysis of OM47 reveals a β-barrel outer mitochondrial membrane protein. (A) The **s**equence of OM47 was retrieved from The Arabidopsis Information Resource (TAIR10, http://www.arabidopsis.org/). OM47 is predicted to belong to the Porin3 superfamily of proteins. (i) Sequences corresponding to loops outside of the membrane are indicated in blue and loops inside the membrane are indicated in green. Sequences corresponding to 18 putative β-strands (amino acids: 33–41, 60–68, 74–84, 96–104, 109–117, 122–130, 133–141, 156–162, 165–173, 18–201, 204–214, 233–243, 249–259, 273–289, 301–309, 324–332, 336–346, and 359–371) are indicated in red. Diagrammatic representation is shown in (Aii). (B) Heatmaps of expression analysis for nuclear-encoded genes encoding mitochondrial and chloroplastic β-barrel proteins (i) across plant development in various organs and (ii) across senescence in leaf tissue. Data are shown relative to maximum expression for each gene. Two different heat maps are shown as the senescence array data transcriptome was carried out on a different platform from the Affymetrix developmental series, and thus had to be normalized individually. (C) Phylogenetic analysis of OM47 along with mitochondrial and chloroplastic β-barrel proteins from *Arabidopsis thaliana* (At), *Physcomitrella patens* (Pp)*, Oryza sativa* (Os), *Zea mays* (Zm), and *Saccharomyces cerevisiae* (Sc) using MEGA5.2.2. Mitochondrial proteins are shaded in orange and chloroplastic proteins are shaded in green. VDAC, voltage-dependent anion channel; TOM40, translocase of the outer mitochondrial membrane; SAM50, sorting and assembly machinery; TOC, translocon at the outer envelope membrane of chloroplasts; OEP, outer envelope protein.

The amino acid sequence of OM47 protein, corresponding to the gene locus At3g27930, was retrieved from The Arabidopsis Information Resource (TAIR10, http://www.arabidopsis.org/). A search for conserved domains using the CDD revealed that OM47 contains the highly conserved mitochondrial outer membrane channel-forming Porin3 family motif (CD07303) within amino acids 183–361 (Fig. 1A) (Marchler-Bauer *et al.*, 2013). The β-strands of the OM47 protein were identified using PRED-TMBB (http://bioinformatics.biol.uoa.gr/PRED-TMBB/) based on a hidden Markov model (Bagos *et al.*, 2004). (Fig. 1A).

Transcript expression analysis of *OM47*, as well as other genes encoding mitochondrial outer β-barrel membrane proteins (i.e. VDAC, TOM40, and SAM50) and chloroplast outer envelope β-barrel proteins (i.e. TOC75, OEP, and TGD) was applied to identify any putative roles these proteins may have during plant development. *OM47* transcript abundance was relatively low throughout most developmental stages; however, it was slightly higher during the early stages of germination and throughout leaf senescence (Fig. 1Bi, Bii).

The amino acid sequences of OM47, VDAC, TOM40, SAM50, OEP37, OEP24, OEP21, OEP80, and TOC75 were retrieved from Arabidopsis, *Physcomitrella patens* (Pp), *Zea mays* (Zm), and *Oryza sativa* (Os) databases using Phytozome 9.0, along with *Saccharomyces cerevisiae* (Sc) VDAC, SAM50, and TOM40 to construct a bootstrapped consensus tree using MEGA 5.2.2 (Tamura *et al.*, 2011). Phylogenetic analysis reveals that orthologues of OM47 and VDAC branch as sister clades from TOM40, with both probably being derived from a common ancestor (Fig. 1C). Although OM47 is related to both VDAC and TOM40, it represents its own distinct clade that is already present in the early land plant *P. patens* and is conserved in higher plants.

### Arabidopsis thaliana *OM47 can functionally replace the VDAC protein in a yeast* (Saccharomyces cerevisiae) *vdac mutant*

**Fig. 2.**
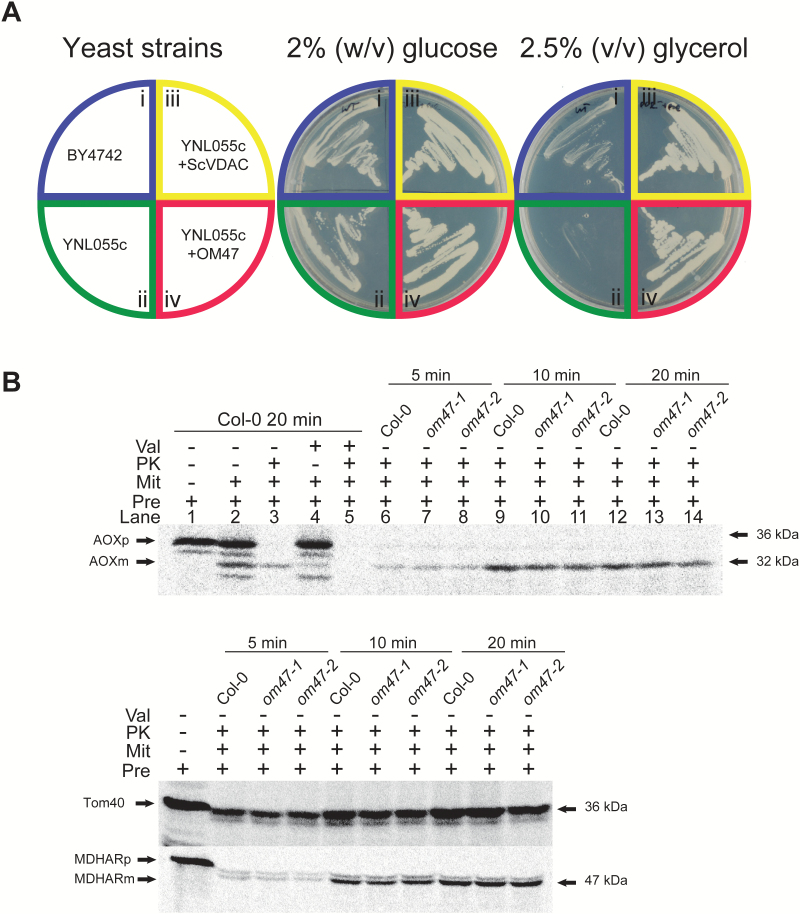
*Arabidopsis thaliana* OM47 can functionally replace yeast VDAC. (A) Complementation assay of the yeast (*Saccharomyces cerevisiae*) ScVDAC protein with OM47. The yeast VDAC deletion strain ΔYNL055c was transformed with the OM47 and ScVDAC. The ΔYNL055c+OM47 complemented strain grew on the medium with glycerol, suggesting that OM47 can functionally replace the ScVDAC protein. ΔYNL055c+ScVDAC was used as a positive control. (B) *In vitro* import assays of precursor proteins into isolated mitochondria from 2-week-old wild-type and *om47* plants. Upper panel: to verify the import assay, the precursor for alternative oxidase (AOXp) was imported into mitochondria isolated from wild-type plants, in the presence and absence of valinomycin, that dissipates the membrane potential across the inner membrane required for protein import into or across the inner membrane. A protease-protected product (mature AOX, AOXm) was detected (lane 3) that was not present in the presence of valinomycin (lane 5). No difference in the amount of protein import between mitochondria isolated from wild-type plants (Col-0) and *om47* mutants could be detected over 20min. Lower panel: the TOM40 and monohydroascorbate reductase (MDAR) precursor proteins were used instead of AOX precursor as in lanes 1 and 6–14 in the upper panel. Val, valinomycin; PK, protease K; Mit, mitochondria, Pre, precursor protein.

As OM47 contains the Porin3 superfamily domain and shares the highest identity and similarity scores with VDAC proteins, it was tested whether OM47 can functionally replace the VDAC protein. A complementation experiment was carried out as described previously using a yeast strain (ΔYNL055c) lacking the VDAC protein (Lee *et al.*, 2009). As anticipated, strains of the wild type (BY4742), ΔYNL055c, ΔYNL055c complemented with the yeast wild-type *VDAC* gene (ΔYNL055c+ScVDAC), or complemented with *OM47* (ΔYNL055c+OM47), all grew well on media with 2% (w/v) glucose (Fig. 2A). Although the BY4742 strain grew well on media with 2.5% glycerol, ΔYNL055c did not grow on the glycerol medium (Fig. 2A) as reported previously (Lee *et al.*, 2009). The ΔYNL055c yeast strain transformed with an *OM47*-containing construct was able to remain viable on media containing glycerol as the sole source of carbon, as observed with the ΔYNL055c strain transformed with Sc*VDAC* (Fig. 2A), suggesting that OM47 can functionally complement yeast VDAC. *In vitro* protein import assays using mitochondria isolated from wild-type plants and *om47* mutants (see below for detailed characterization) did not display any effects on protein import ability (Fig. 2B). The rate of import of the alternative oxidase (AOX) via the general import pathway and the dual targeted protein monodehydroascorbate reductase (MDHAR) displayed no change in protein import as determined by the amount of mature protein in mitochondria isolated from *om47* plants compared with mitochondria isolated from wild-type Arabidopsis plants. Import of TOM40 via the SAM pathway as determined by the amount of proteinase K (PK)-protected protein similarly showed no difference (Fig. 2B). From this, it was concluded that OM47 does not play a role in protein import unlike the other two β-barrel proteins TOM40 and SAM50. Taken together with the ability of OM47 to complement a *vdac* mutant from yeast, this suggests a likely involvement of OM47 in the transport of small molecules or metabolites.

### *Confirmation and characterization of T-DNA insertional knock-out lines for* Arabidopsis thaliana *OM47*

**Fig. 3.**
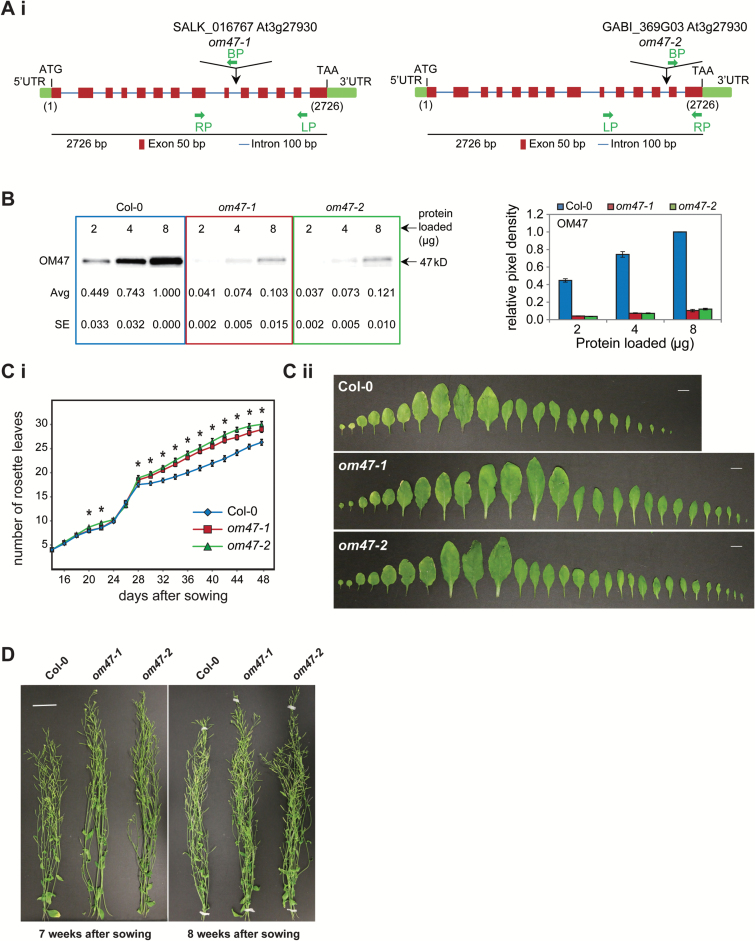
Confirmation and characterization of T-DNA insertional knock-out lines for *Arabidopsis thaliana* OM47. (A) Diagram illustrating the positions of the T-DNA inserts in (i) *om47-1* (SALK_016767) and (ii) *om47-2* (GABI_369G03) lines of the *A. thaliana OM47* (At3g27930) gene. Also shown is the length (bp), UTRs (untranslated regions), ATGs (start codon), TAAs (stop codon), exons (red squares), and introns (blue lines) of the *OM47* (At3g27930) gene (see Supplementary Fig. S1 for the exact site of T-DNA inserts). LP, RP, and BP represent the primers used for screening the T-DNA insertions (see Supplementary Table S1 for specific primer sequences). (B) SDS–PAGE (left panel) and quantified relative pixel density (right panel) showing OM47 proteins in mitochondria isolated from 2-week-old *A. thaliana* wild type (Col-0), *om47-1* (SALK_016767), and *om47-2* (GABI_369G03). The amounts of mitochondrial protein loaded and apparent molecular mass of the protein detected (47kDa) are indicated. Serial dilutions of mitochondrial proteins were used to ensure linearity of detection, and immunodetection was carried out in biological triplicate, with numbers giving averages (avg) and SEs. (C) (i) The two *om47* mutant lines had an increased number of leaves from ~8 d after sowing when compared with the wild type (Col-0) which was maintained through later stages of development. (ii) A series of developmental stages of all leaves from the 7-week-old wild type and *om47* mutants reveals the increased number of leaves in the two mutant lines. In addition, the oldest leaves of the wild type show an earlier onset in senescence when compared with the mutants. (D) At late developmental stages (7 and 8 weeks after sowing), both *om47* mutant lines had a higher inflorescence than the wild type.

Two independent T-DNA insertion lines for *OM47* (At3g27930) were characterized. PCR screening and genomic DNA sequencing of these lines revealed that both *om47-1* and *om47-2* have inserts within the intron regions (Fig. 3Ai, 3Aii; Supplementary Fig. S1). To confirm protein abundance, a polyclonal antibody was raised against the full-length OM47 protein. SDS–PAGE and immunodetection on total mitochondrial protein isolated from 3-week-old wild-type (Col-0) and *om47* mutant plants revealed an ~90% decrease in OM47 protein abundance in the two T-DNA lines (Fig. 3B), showing that the T-DNA inserts in the introns effectively reduced the amount of the OM47 protein.

Phenotypic analysis of *om47* mutant lines grown on MS media, supplemented with or without 3% (w/v) sucrose, was carried out in accordance with parameters as described previously (Boyes *et al.*, 2001). For lines grown on both types of MS media, no significant difference in phenotype was observed (Supplementary Fig. S2A, B). Soil-based phenotyping initiated at 14 d indicated that all lines reached stage 1.04 (four rosette leaves) simultaneously (Supplementary Fig. S2C). Col-0 and *om47-1* reached the first flower open stage (6.0) at day 28; *om47-2* was significantly (*P*≤0.05) delayed by 2 d (day 30) (Supplementary Fig. S2C). The significantly (*P*≤0.05) greater number of leaves in both mutant lines indicates an increased leaf production before phase transition (Fig. 3C). Furthermore, although *om47-2* initially showed on average a significantly lower inflorescence height compared with Col-0 and *om47-1* up until day 31 (Supplementary Fig. S2D), a subsequent increase in inflorescence growth of *om47-2* resulted in a higher inflorescence in both *om47-1* and *om47-2*, from day 50 when compared with the wild type (Fig. 3D; Supplementary Fig. S2D, E). Finally, a significant extension (*P*≤0.05) of the total flowering time by ~4 d and increased maximum rosette radius was observed in both mutant lines (Supplementary Fig. S2C–E).

### Arabidopsis thaliana om47 *mutants display an extended growth period phenotype that prolongs photo-assimilation and starch accumulation*

**Fig. 4.**
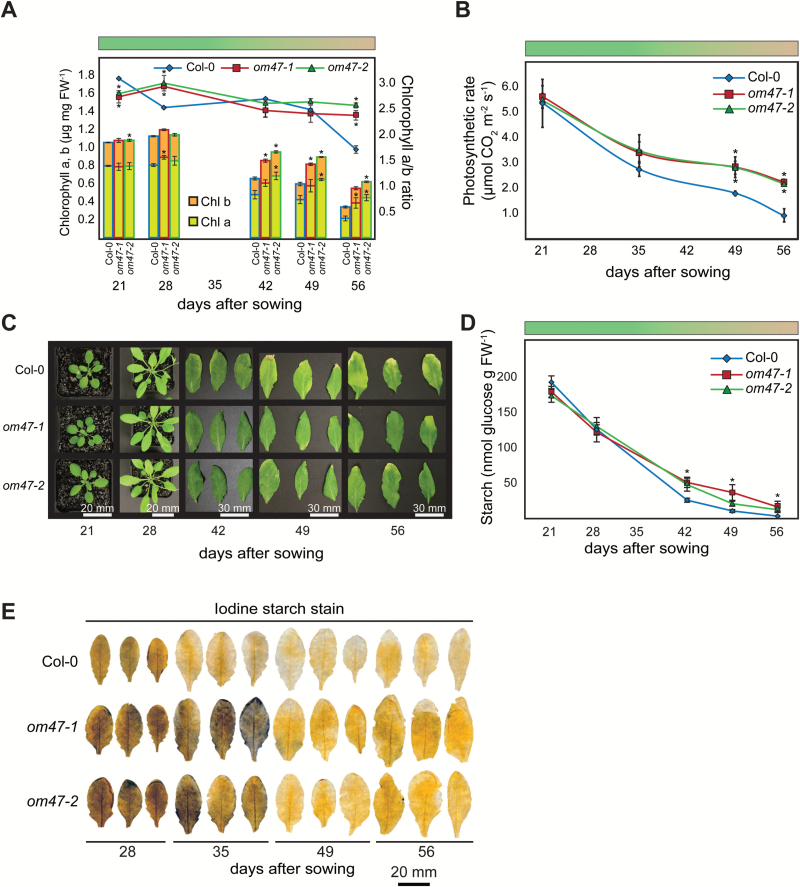
*Arabidopsis thaliana om47* mutants display a stay-green phenotype during senescence that prolongs photoassimilation and starch accumulation. (A) The chlorophyll, Chl *a*, and Chl *b* concentrations and the Chl *a*/*b* ratio of the *A. thaliana* wild type (Col-0) and *om47* mutants over 56 d under standard (16/8h photoperiod) growth conditions. The coloured bar represents the stage of senescence throughout the experiment (*n=*5; data are given as averages; error bars represent ±SE). *Significant differences (*P*≤0.05) compared with the wild type at each time point within treatments as determined by Student’s *t*-test. (B) Photosynthetic rate of *A. thaliana* wild type (Col-0) and *om47* mutants over 56 d under standard (16/8h photoperiod) growth conditions. The coloured bar represents the stage of senescence throughout the experiment (*n =* 4; data are given as averages; error bars represent ±SE). *Significant differences (*P*≤0.05) compared with the wild type as determined by Student’s *t*-test. (C) Representative rosette and leaf images of *A. thaliana* wild type (Col-0) and *om47* mutants over 56 d under standard (16/8h photoperiod) growth conditions. The bar indicates the scale. (D) Starch concentration and (E) iodine starch stains of the *A. thaliana* wild type (Columbia-0) and *om47* mutants over 56 d under standard (16/8h photoperiod) growth conditions at the end of the light period (EOLP, ZT16). The coloured bar represents the stage of senescence throughout the experiment (*n=*5; data are given as averages; error bars represent ±SE). *Significant differences (*P*≤0.05) compared with the wild type as determined by Student’s *t*-test. The bar indicates scale.

As *om47* lines consistently exhibited 3–4 additional rosette leaves compared with the wild type, close biochemical and physiological monitoring of leaf development across the mid (21 d post-germination) to late (56 d post-germination) developmental stages was carried out. There was a decrease in chlorophyll after day 28 in the wild type and both *om47* lines, but the decline in the *om47* mutants was less pronounced (Fig. 4A) Furthermore, leaf gas exchange measurements were carried out in wild-type and *om47* leaves. The photosynthetic rate in *om47* mutants was comparable with that of the wild type at 21 d (Fig. 4B). From then on, there was a steady reduction during ageing that paralleled chlorophyll concentrations (Fig. 4A, B). As with the chlorophyll concentration, the reduction in photosynthetic rate was significantly (*P*<0.05) less in *om47* mutants compared with wild-type plants, with a 1.5-fold difference at 49 d and 2-fold by 56 d (Fig. 4B). In agreement with these data, senescence seemed to occur earlier for wild-type than for *om47* mutant plants (Fig. 4C). The similarity of this trend was observed in other physiological parameters as well, including transpiration, stomatal conductance, and dark respiration (Supplementary Fig. S3). Additionally, starch concentration was quantified in wild-type and *om47* mutant plants at the end of the light period throughout development. Not surprisingly, starch concentration in the wild type and *om47* mutants was reduced as leaf ageing progressed (Fig. 4D, E). However, starch concentration in both *om47* mutant plants was significantly (*P*≤0.05) greater than in the wild type at the three latest points (42, 49, and 56 d).

### Arabidopsis thaliana om47 *mutants delay chlorophyll breakdown during dark-induced senescence*

**Fig. 5.**
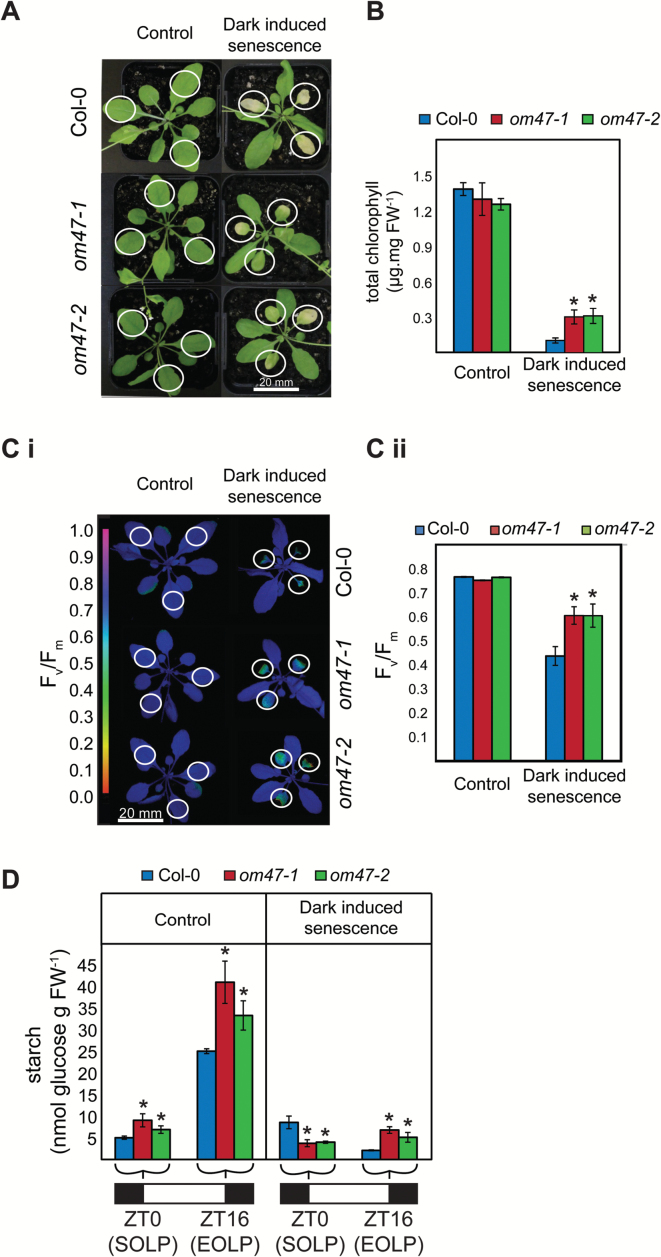
Phenotypes in *Arabidopsis thaliana om47* mutants prolong chlorophyll breakdown during (attached leaf) dark-induced senescence. (A) Representative rosette images for 29-day-old *A. thaliana* wild type (Col-0) and *om47* mutants under controlled (16h light/8h dark photoperiod) growth conditions and 8 d of dark-induced senescence (24h dark) conditions. Only leaves 5, 6, and 7 were either dark acclimated or used as controls as indicated by circles (see also Ci). The bar indicates scale. (B) Chlorophyll concentration of emerging leaves 5, 6, and 7 (indicated by circles in A) from 29-day-old *A. thaliana* wild type (Col-0) and *om47* mutants under control (16h light/8h dark photoperiod) and after 8 d of dark-induced senescence (24h dark photoperiod) conditions (*n=*5; data are given as averages; error bars represent ±SE). *Significant differences (*P*≤0.05) compared with the wild type at each time point within treatments as determined by Student’s *t*-test. (C) PSII maximum efficiency (*F*_v_/*F*_m_) images (i) and quantifications (ii) illustrating photosynthetic capacity of three covered or uncovered leaves (indicated by circles) from 29-day-old *A. thaliana* wild type (Columbia-0, Col-0) and *om47* mutants under control (16h light/8h dark photoperiod) and 8 d of dark-induced senescence (24h dark photoperiod) conditions. (*n=*4; data are given as averages; error bars represent ±SE). *Significant differences (*P*≤0.05) compared with the wild type in each treatment as determined by Student’s *t*-test. (D) Starch concentration of three uncovered leaves in controlled (16h light/8h dark photoperiod) growth conditions and three covered leaves in 8 d of dark acclimation (24h dark photoperiod; attached leaf) from 29-day-old *A. thaliana* wild type (Columbia-0, Col-0) and *om47* mutants grown under the standard (16/8h photoperiod) growth conditions at the start of the light period (SOLP, ZT0) and at the end of the light period (EOLP, ZT16). Measurements were carried out in biological triplicates and data are given as averages for pools of five plants per genotype; error bars represent ±SE. *Significant differences (*P*≤0.05) compared with the wild type at each time point within treatments as determined by Student’s *t*-test.

**Fig. 6.**
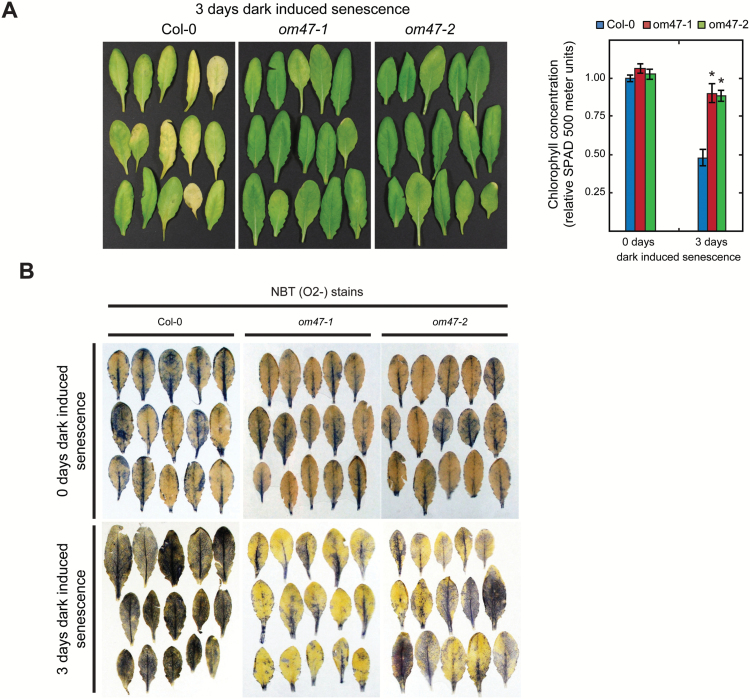
*Arabidopsis thaliana om47* mutants display a stay-green phenotype, and have altered reactive oxygen species accumulation in a dark-induced senescence assay on detached leaves. (A) Individual leaves from *A. thaliana* wild type (Col-0) and *om47* mutants detached from 4-week-old plants and covered for 3 d to induce the senescence process. The right-hand panel shows relative senescence-related chlorophyll breakdown of *A. thaliana* wild type (Col-0) and *om47* mutants before (4-week-old plants, 0 d of dark-induced senescence) and after (3 d of dark-induced senescence) detachment from 4-week-old plants as quantified by using the SPAD 500 chlorophyll meter. Values are relative to the wild type at 0 d of dark-induced senescence. Leaves were detached and placed inside lightproof plates. Five leaves per plant and three plants per genotype were sampled (*n*=3; data are given as averages; error bars represent ±SE). *Significant differences (*P*≤0.05) compared with the wild type at each growth stage as determined by Student’s *t*-test. (B) Reactive oxygen species in the form of O_2_^−^ from individual leaves of *A. thaliana* wild type (Col-0) and *om47* mutants before (4-week-old plants, 0 d of dark-induced senescence) and after (3 d of dark-induced senescence) detachment from 4-week-old plants.

To investigate further the function of OM47 during development, dark-induced senescence experiments were carried out. Individual leaves from 3-week-old wild-type (Col-0) and *om47* mutants were covered with aluminium foil for a total of 8 d to trigger dark-induced senescence (Fig. 5). Furthermore, individual leaves were excised from 4-week-old Col-0 and *om47* mutants, placed in Petri dishes with moist blotting paper, and covered for a total of 3 d to trigger dark-induced senescence (Fig. 6).

Consistent with the higher chlorophyll concentration observed during development (Fig. 4) in the *om47* lines, chlorophyll breakdown during dark-induced senescence was reduced in *om47-1* and *om47-2* compared with the wild type after 8 d of dark-induced senescence treatment in attached leaves (Fig. 5A, B). Following treatment, the chlorophyll concentrations in the covered leaves of *om47* mutants were significantly (~2.5 times, *P*≤0.05) greater than in the wild type (Fig. 5B) and displayed higher photosynthetic efficiency (*F*_v_/*F*_m_) (Fig. 5C). In addition, wild-type *F*_v_/*F*_m_ was reduced from 0.75 to 0.45, while it decreased for *om47* mutants to only 0.6 (Fig. 5Cii). The starch concentration in *om47* mutants was significantly greater than in Col-0 at the end of the light period (EOLP) in both control and dark-induced senescence (Fig. 5D) (*P*≤0.05).

The chlorophyll concentration was measured non-destructively (using a SPAD 500 chlorophyll meter) in detached leaves before (day 0) and after 3 d of acclimation to darkness. Following dark acclimation, chlorophyll concentration dropped by 50% in wild-type leaves while it was reduced by only 10% in the two *om47* mutant plants (Fig. 6A). During dark-induced senescence, large quantities of ROS such as superoxide, hydrogen peroxide, hydroxyl radicals, and singlet oxygen are produced (Sedigheh *et al.*, 2011). Cellular ROS production (as superoxide anions, O_2_^−^) in leaves of the wild type and *om47* lines was visualized when stained with NBT. Before dark acclimation, there were no observable differences in steady-state levels of ROS in either Col-0 or *om47* plants (Fig. 6B). Following the 3 d dark treatment of detached leaves, production of ROS in *om47* plants was less than the level seen in Col-0 leaves, indicating a lower level of stress-induced ROS accumulation in *om47* mutants (Fig. 6B).

**Fig. 7.**
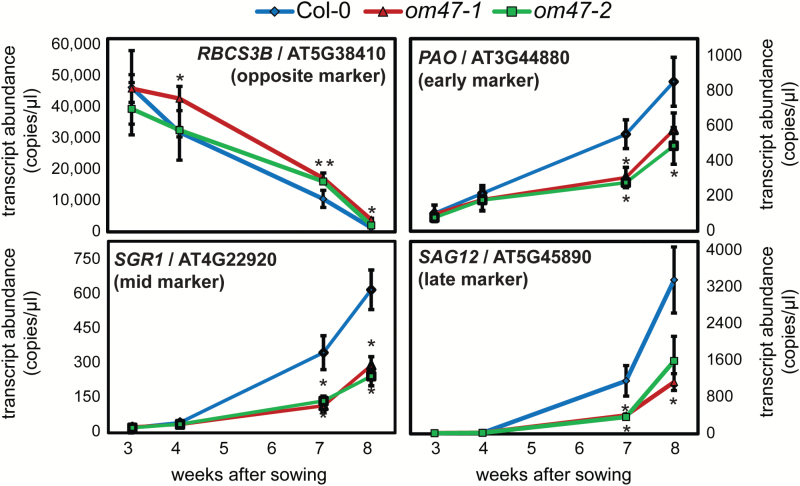
Digital RT–PCR of senescence marker genes in *Arabidopsis thaliana om47* mutants during natural senescence. Quantification of transcript abundance by digital droplet RT–PCR for *RUBISCO SMALL SUBUNIT 3B* (*RBCS3B*; At5g38410), (*PHEOPHORBIDE A OXYGENASE* (*PAO*; At3g44840), *STAY-GREEN 1* (*SGR1*; At4g22920), and *SENESCENCE-ASSOCIATED GENE 12* (*SAG12*, At5g45890) from leaves of *A. thaliana* wild-type (Columbia-0, Col-0) and *om47* mutants at 21, 28, 49, and 56 d after germination. The decrease in abundance of the transcript of *RBCS3B* (declining under senescence) was used as a senescence-repressed marker, while PAO (early senescence marker), SGR1 (middle senescence marker), and SAG12 (late senescence marker) were included as senescence-induced markers (*n=*3; data are given as averages; error bars represent ±SE). *Significant differences (*P*≤0.05) compared with the wild type at each time point within treatments as determined by Student’s *t*-test.

To determine whether the differential regulation of the chlorophyll concentration between the wild type and the *om47* mutants was linked to the process of senescence, the transcript abundance of typical senescence marker genes was evaluated (Brouwer *et al.*, 2014). As expected, transcript abundance of the gene encoding the small subunit of 1, 5-ribulose bisphosphate carboxylase oxygenase (At5g38410; *RBCS3B*) decreased in abundance from 3-week-old leaves to 8-week-old leaves of both wild-type and *om47* plants (Fig. 7). There was no significant difference in transcript abundance for *RBCS3B* in both *om47* mutant lines compared with the wild type when plants were growing for 3 weeks. Although the transcript abundance of *RBCS3B* showed a consistent decline in all three genotypes with age, it was significantly higher than in the wild type in the *om47-1* line at 4 weeks, and for both *om47* lines at 7 and 8 weeks (Fig. 7). Furthermore, expression analyses of other marker genes for senescence, such as *PHEOPHORBIDE A OXYGENASE* (*PAO*, early senescence marker), *STAY-GREEN 1* (*SGR1*, mid senescence marker), and *SENESCENCE-ASSOCIATED GENE 12* (late senescence marker) (Breeze *et al.*, 2011; Thomas and Ougham, 2014), all increased in expression over time, indicating that the developmental process of senescence had been initiated (Fig. 7). For each of these senescence marker genes, the transcript abundance was, however, significantly higher (*P*<0.05) in wild-type plants compared with the *om47* mutants at 7 and 8 weeks (Fig. 7). These expression patterns support the observed delayed senescence phenotype of the two *om47* mutants as already observed by the physiological and biochemical characterizations.

**Fig. 8.**
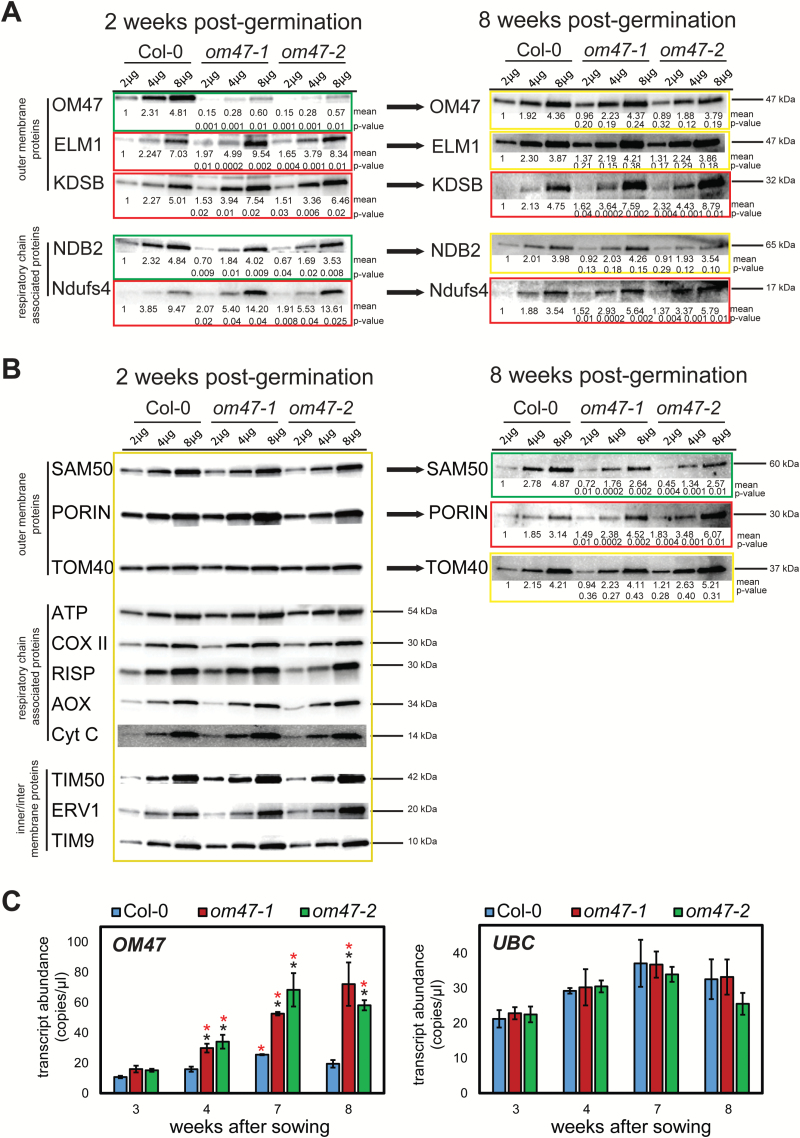
Analyses of the abundance of mitochondrial proteins. Immunological analyses of protein isolated from mitochondria of 2-week-old and 8-week-old *Arabidopsis thaliana* wild type (Columbia-0, Col-0) and *om47* mutants probed with various antibodies raised to mitochondrial proteins. (A) Proteins that were found to change significantly in abundance between mitochondria isolated from 2-week-old wild-type plants and *om47* mutants are shown. (B) Analyses of the proteins that did not significantly change in abundance between mitochondria isolated from 2-week-old wild-type plants and *om47* mutants are shown. Yellow boxes indicate no significant difference, while red and green boxes indicate a significant increase and decrease, respectively, in abundance in mitochondria isolated from the wild type and *om47* mutants. *Significant differences (*P*-value shown) compared with the wild type at each time point within treatments as determined by Student’s *t*-test. The apparent molecular mass is indicated. (C) Digital droplet RT–PCR transcript abundance of *OM47* from leaves of *A. thaliana* wild type (Columbia-0, Col-0) and *om47* mutants at 3, 4, 7, and 8 weeks after germination (*n=*3; data are given as averages; error bars represent ±SE). *Significant differences (*P*≤0.05) of each line compared with samples at 3 weeks as determined by Student’s *t*-test. Ubiquitin (At5g25760) was used as a control to ensure equal RNA input and for normalization of transcript abundance.

To investigate further the stay-green phenotype in *om47* mutants, mitochondria were purified from 2-week-old *om47* mutants and wild-type plants, and immunodetection analysis was carried out using a number of antibodies to known mitochondrial proteins (Fig. 8). As previously shown (Fig. 3), OM47 protein was reduced by ~85–90% in abundance in *om47* mutants compared with the wild type (Col-0) (Fig. 8A). NDB2 [an alternative external NAD(P)H dehydrogenase located on the mitochondrial inner membrane] also showed an ~25% lower abundance in *om47* plants compared with the wild type. Three proteins that were examined were significantly more abundant in *om47* mutants: the outer mitochondrial membrane protein ELM1 (ELONGATED MITOCHONDRIA 1, located in the mitochondrial outer membrane, At5g22350) (Arimura *et al.*, 2008), a subunit of complex I, Ndufs4 (NADH:UBIQUINONE OXIDOREDUCTASE FE-S PROTEIN4, At5g67590) (Meyer *et al.*, 2009), and KDSB (CMP-KDO SYNTHETASE, At1g53000) (Duncan *et al.*, 2011) (Fig. 8A). A variety of other mitochondrial proteins, including the β-barrel protein family members Porin, TOM40, and SAM50 (Duncan *et al.*, 2011), did not change significantly in abundance between the wild type and mutants (Fig. 8B) in mitochondria isolated from 2-week-old plants.

Mitochondria were also isolated from 8-week-old plants and the yield was noticeably reduced on a fresh weight basis compared with 2-week-old samples (data not shown). Similar immunodetection analyses using the antibodies to proteins that displayed differences in abundance from mitochondria isolated from 2-week-old plants revealed that by this time, the abundance of OM47 was in fact equal in all three genotypes (Fig. 8A). The decrease in the abundance of NDB2 was also no longer seen for the 8-week-old plants (Fig. 8A). Analysis of other outer membrane β-barrel proteins revealed that PORIN increased in abundance (*P*<0.01), while SAM50 was significantly decreased in abundance (*P*<0.01) in the *om47* samples compared with the wild type. There was no difference in the abundance of TOM40 (Fig. 8B).

In order to investigate the underlying mechanism involved in the significant increase in OM47 protein in the *om47-1* and *om47-2* lines at 8 weeks, transcript abundance of OM47 was examined in 3-, 4-, 7-, and 8-week-old leaf tissue. A clear increase in transcript abundance of *OM47* in the *om47* lines was observed after the 3 week time point, with a 2- to 4-fold higher expression seen compared with the wild type (Fig. 8C). These increases in transcript abundance were also paralleled on the protein level (Supplementary Fig. S4). Thus, a developmental compensation occurs to increase the abundance of OM47 protein, which was at 10–15% of the wild type in mitochondria isolated from 2- to 4-week old *om47* mutants, to wild-type levels at 8 weeks.

## Discussion

In this study, the role of a novel plant-specific β-barrel protein, OM47, was investigated. Based on the observed ability to complement a *vdac* mutant in yeast, and the fact the absence of OM47 had no apparent effect on the import of precursor proteins into isolated mitochondria, it was concluded that OM47 plays a role in the transport of metabolites, akin to VDAC. This is consistent with OM47 branching from the VDAC lineage at least 400 million years ago based on the presence of an OM47 orthologue in *P. patens* (Schaefer and Zryd, 2001). Two independent T-DNA lines obtained for the gene (At3g27930) encoding OM47 display an ~90% reduction in the amount of OM47 protein from mitochondria isolated from 2-week-old seedlings. Subsequent analyses of the physiological consequences revealed an apparent delayed senescence in both attached and detached leaves upon dark-induced senescence in the two *om47* mutants. This delay in senescence was evidenced by a significantly attenuated up-regulation of typical senescence-induced marker genes (*SAG12*, *PAO*, and *SGR1*), a delay in chlorophyll breakdown, and a subsequent maintenance of a higher *F*_v_/*F*_m_ value, that resulted in a greater amount of starch being present after the various treatments.

The decreased abundance of OM47 in the two T-DNA insertion lines at 2 weeks was also accompanied by an increase in the abundance of three other mitochondrial proteins, namely ELM1, KDSB (Duncan *et al.*, 2011), and Ndufs4, a subunit of complex I (Meyer *et al.*, 2009). A decrease in the abundance of an alternative NAD(P)H dehydrogenase, NDB2, was observed (Elhafez *et al.*, 2006). Unexpectedly at week 8, the amount of OM47 protein in mitochondria isolated from *om47* mutants was the same as observed in mitochondria isolated from wild-type plants. This recovery of OM47 protein was achieved by a >3-fold increase in transcript abundance of *OM47* in the *om47* lines compared with the wild type. This surprising up-regulation, likely to be a compensatory mechanism, strongly reinforces the idea that OM47 plays a major role during leaf senescence. Furthermore, the recovery of OM47 to wild-type levels was paralleled by similar increases in ELM1 and NDB2, that were at wild-type levels at week 8, although KSDB and the complex I subunit, Ndufs4, remained elevated. Importantly, the recovery in OM47 protein abundance was accompanied by changes in other outer mitochondrial membrane β-barrel proteins, namely SAM50 decreased and VDAC (Porin) increased. Thus, overall there was a complex series of events occurring, whereby the factors driving the recovery of the abundance of OM47 protein, based on elevated transcript abundance, also impacted the abundance of other proteins. Note that alterations in the abundance of the other proteins, especially the outer membrane proteins, may be occurring at a post-transcriptional level, as previously it has been documented that changes in the abundance of other membrane proteins or proteins associated with protein import into mitochondria occurred without any changes in transcript abundance of the genes encoding these proteins (Howell *et al.*, 2006; Lister *et al.*, 2007; Narsai *et al.*, 2011; Law *et al.*, 2012).

The decrease in the abundance of OM47 in the mutant lines can be compensated for by elevating transcript abundance, probably because the presence of both T-DNA insertions in an intron allows for the production of an authentic OM47 transcript after splicing. However, this compensation was not sufficient or fast enough to restore normal function as a distinct delayed senescence phenotype could be observed in the *om47* mutants. The observation of this altered phenotype in dark-induced senescence assays was consistent with the increase in the number of rosette leaves in the *om47* mutants. Essentially the *om47* plants showed an extended vegetative growth period, producing more leaves and increased biomass before bolting. The most visible sign of this delayed senescence was the delay in chlorophyll breakdown, evidenced in both *om47* mutants. It has been established in a number of plant systems that mitochondria persist long after chloroplasts are undergoing disassembly (Keech *et al.*, 2007). This suggests a central role for mitochondria to support cell energy functions, to ensure all nutrients and molecules are recovered during senescence before cell death. As such it would be expected that the transport of various metabolites through the outer membrane would be essential to execute the programmed process of senescence. Given the uniqueness of leaf senescence to plants, it may not be surprising that a protein involved in facilitating metabolites into and out of mitochondria may have specialized in the plant lineage.

**Fig. 9.**
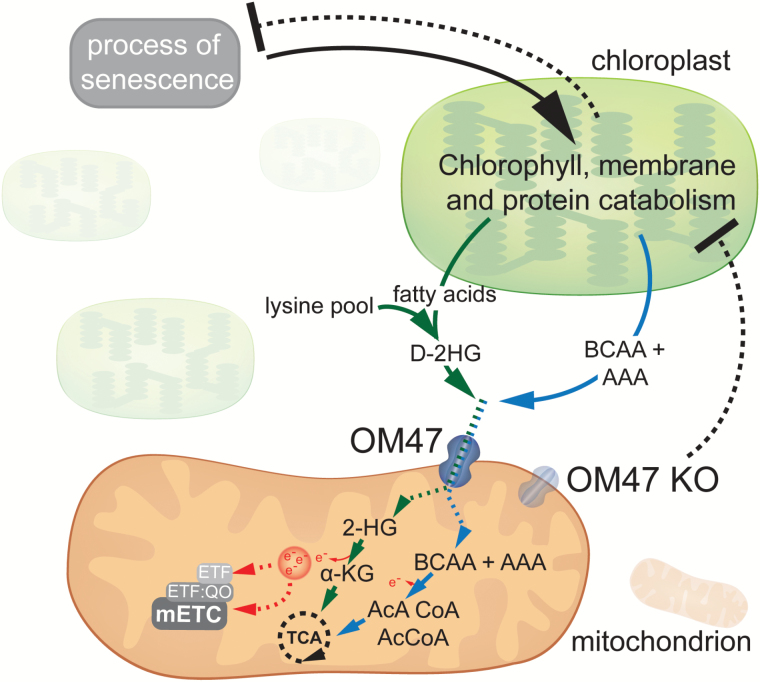
Model describing the putative role of OM47 and its effect on the process of senescence as observed through the use of T-DNA insertion lines. Chloroplast disassembly leads to the release of BCAAs, AAAs, and 2-HG, which in turn can provide reducing equivalents and carbon skeletons to support mitochondrial functions (see Discussion for details). Plain lines/arrows represent known molecular mechanisms while dotted lines/arrows indicate putative mechanisms. AAA, aromatic amino acid; BCAA, branched chain amino acid; 2-HG, 2-hydroxyglutarate; AcCoA, acetyl-CoA; AcACoA, acetoacetyl-CoA; α-KG, α -ketoglutarate; mETC, mitochondrial electron transport chain; TCA, tricarboxylic acid cyle.

The specific metabolites that may be transported by OM47 are not yet defined. VDAC proteins are reported to be able to transport molecules ranging up to 4000Da (Colombini, 2012). OM47 might be transporting a number of metabolites and, in its absence, the metabolic readjustments during senescence might be retarded, to result in metabolic feedback on the process of senescence, leading to a delayed breakdown in chlorophyll and disassembly of chloroplasts. Also, OM47 could be specifically involved in the breakdown of chlorophyll. From current knowledge, there are two types of specific metabolites for which OM47 might mediate transport (Fig. 9). The first option is that OM47 is involved in transporting 2-hydroxyglutarate (2-HG), a metabolite that can derive from lysine catabolism as well as from fatty acid degradation (Araujo *et al.*, 2010; Engqvist *et al*., 2011). Once imported into the mitochondrion, 2-HG is oxidized to α-ketoglutarate, which can then enter the tricarboxylic acid (TCA) cycle. Such reactions also lead to the production of reducing equivalents that could provide electrons to the mitochondrial electron transport chain (mETC), which in turn generates ATP to support the senescence-associated catabolic processes. The second option is that OM47 participates in channelling branched chain amino acids (BCAAs) and aromatic amino acids (AAAs), mostly arising from chloroplast degradation, into the mitochondrion. Indeed, it has been reported that BCAAs and AAAs can also provide reducing equivalents to the mETC via the electron transfer flavoprotein complex/ubiquinone oxide reductase under senescence and starvation situations (Ishizaki *et al.*, 2006; Araujo *et al.*, 2010; Peng *et al.*, 2015). Furthermore, this reaction produces acetoacetyl- and acetyl-CoA, which can feed into the TCA cycle and further support metabolic reactions (Fig. 9). Although such metabolic schemes are legitimate, we cannot currently rule out other types of metabolites being transported via OM47. Regardless, it is likely that OM47 channels pivotal metabolites, and that in its absence the metabolic readjustments during leaf senescence are retarded. This in turn results in a metabolic feedback on the process of senescence, as shown by the delayed breakdown in chlorophyll and disassembly of chloroplasts (Fig. 9). It is worth noting that these processes may lead to altered signalling as a result of altered metabolite pools. The observed changes in starch levels in the *om47* mutants may affect sugar signalling from organelles (Lastdrager *et al.*, 2014). The retarded breakdown of chlorophyll may lead to an accumulation of intermediates of chlorophyll breakdown. As various haem or pigment biosynthetic intermediates are known to have signalling properties (Chan *et al.*, 2016), it is not unexpected that any alterations in chlorophyll breakdown may have implication for signalling.

In summary, the transport of molecules into and out of mitochondria may have both direct and indirect effects on the overall metabolic homeostasis during leaf senescence. If perturbed, as in the *om47* mutants, this results in the delay of the whole process.

## Supplementary data

Supplementary data are available at *JXB* online.

Figure S1. T-DNA insertion position as determined by sequencing for the T-DNA lines used in this study.

Figure S2. Plate-based development stages of *Arabidopsis thaliana om47* mutants.

Figure S3. *Arabidopsis thaliana om47* mutants have enhanced gas exchange parameters during senescence.

Figure S4. Relative protein abundance of OM47 in mitochondria isolated from *om47-2* mutant plants at different developmental stages.

Table S1. List of primers used in this study.

Table S2. List of antibodies used in this study.

Supplementary Data
