# Supplementary material for: Characterization of a novel β-barrel protein (AtOM47) from the mitochondrial outer membrane of Arabidopsis thaliana
Source: J Exp Bot. 2016 Oct 6;67(21):6061–75. doi: 10.1093/jxb/erw366 (PMC5100019; doi:10.1093/jxb/erw366)
Supplement: Supplementary Data [file supp_67_21_6061__index.html]

Characterization of a novel β-barrel protein (AtOM47) from the mitochondrial outer membrane of Arabidopsis thaliana — Characterization of a novel β-barrel protein (AtOM47) from the mitochondrial outer membrane of Arabidopsis thaliana — Supplementary Data 

# Characterization of a novel β-barrel protein (AtOM47) from the mitochondrial outer membrane of *Arabidopsis thaliana*

## Supplementary Data

Data files

- supplementary\_figures\_S1\_S4\_Tables\_S1\_S2.pdf - Supplementary Data
