# Supplementary material for: Characterization of a novel β-barrel protein (AtOM47) from the mitochondrial outer membrane of Arabidopsis thaliana
Source: J Exp Bot. 2016 Oct 6;67(21):6061–75. doi: 10.1093/jxb/erw366 (PMC5100019; doi:10.1093/jxb/erw366)
Supplement: Supplementary Data [file supp_erw366_supplementary_figures_S1_S4_Tables_S1_S2.pdf]

```
      ---------- ---------- ---------- ---------- ---------- ---------- ---------- ---------- atattcaaac gtgaagcaaa
      agcagaagcg agactatcgc cgagagctct cactaactat tgaattcatc gaagaagctc tctcgttgtc gcagaatctc ttcttctccg ttctagaaca
1     ATGGGGAATG CTCTTGTCAA AAAAGAACCA CCGCCTCCGG TGGTACTCGT TCCTCCGCTC TTTGATTACC CTCCTCTCTC CGCCCGCACT AGgtctacca
       M  G  N   A  L  V   K  K  E  P   P  P  P   V  V  L   V  P  P  L   F  D  Y   P  P  L   S  A  R  T
101   tctcctcttc ttctccgatc tgatcctcat actttcaatt tgttagaatc gcgtgatttc gaagtagttt ttgtataacc taatctaatc aaatagatcg
201   attctgattt ataggttaaa ttgtaattgt gcgattgtga gaaattgttg ttgttgttga tgcagGATGT TGGAATCATC ATATAATTTG TTGTTTGGGA
                                                                           R  M   L  E  S   S  Y  N  L   L  F  G
301   AGCTTGCATT GAGATGTCTA TTTGAGGATT ACTTTGAAGA AGCTAATCGA TTCACGGGCA AGTTTTTGTT GAAGCCTACT GATGATCCTC ATGTGGATTT
       K  L  A   L  R  C  L   F  E  D   Y  F  E   E  A  N  R   F  T  G   K  F  L   L  K  P  T   D  D  P   H  V  D
401   GGTTGCATCT gtatccaatt ttgaaatctc ttttgtgtta tttagtttct atatagtgat gattcgagaa ttttctctaa atggatgtat tagggatatg
      L  V  A  S
501   tagaaaatca gaattttcct taatagtcta aagGTTTCGG GTGCTGTAGA TGGTAGAGTG GAAGGAGATT TTGTTGGGAA TGCGGAGTTT CGCTGGCAAA
                                          V  S   G  A  V   D  G  R  V   E  G  D   F  V  G   N  A  E  F   R  W  Q
601   Ggtatagatt ctttagtttt taatctgaaa gattacaagt agttgttgga gaattggaat actgattagt ttgtttttga taaatatgca gTGATGTTGA
                                                                                                           S  D  V
701   TGATCCTCAT ACTTTTGTTG ATCTCTCTGT GTCAACCTCG AATCCgtaaa ctttgtgcta cttttgatat attcttggag gttatgttca ttacagtagt
      D  D  P  H   T  F  V   D  L  S   V  S  T  S   N
801   ttaatgtttt gaaattgttt gttttgtttg tcatcatcat taagGGTTCT TCTAATGAGG TCTTCTGCTT ACTATCCTAA ATATGGAATC GGGGCATTTG
                                                 P  V   L  L  M  R   S  S  A   Y  Y  P   K  Y  G  I   G  A  F
901   CGGTCTACCC TTTGATTTCA AAAATTACgt aagtcgtatc taggtgtgta ccctgttgtt atatttagat tagatgtaat attgtgaggg tgaatatatg
       A  V  Y   P  L  I  S   K  I
1001  ttgtctctgt agTGGAAAAT CATCTGAAGA ATATAGAATC ATGGGGTTGA GATATGGCTC AACGAATTTG TCTGTCGGAG CTACTGTCAC TCCTTTTAGT
                   T  G  K   S  S  E   E  Y  R  I   M  G  L   R  Y  G   S  T  N  L   S  V  G   A  T  V   T  P  F  S
1101  Ggtaagcgca taaagttgct ctgtgaattc acgttgtttt cttttggaca caatgctgac cctttgattc ttcaattcag CGAATAACGA ATTGCCAAAG
                                                                                             A  N  N   E  L  P  K
1201  CATGCATGGC TTGTAAGCAA GATGGGAAGT CTTACAGTAG GAGTACAATA TGAGCCGCTA Cgtgagttag tttacattgt tttatttctg ttgaacagat
       H  A  W   L  V  S   K  M  G  S   L  T  V   G  V  Q   Y  E  P  L
1301  tttacgcatc acatgtggaa actagtatct ataaattatg tgattgaaag aaagtgagca tcaacaattg ttattggatt ttgttttagA TGGAAGCAAA
                                                                                                        H  G  S  K
1401  GATTTGGCAA AGTACACAGA CCCAAGAAAC TGGAGTTGTG CTGCTGGCTA TGGAGTAGGG TCACAAAGCC CCTTGACTCC TTCTTTTAAC ATTGGCATTG
       D  L  A   K  Y  T   D  P  R  N   W  S  C   A  A  G   Y  G  V  G   S  Q  S   P  L  T   P  S  F  N   I  G  I
1501  AACTAGCAAG AAGCTCTCAG gtgattcttt taaaatccgt gtagatgaaa acatgttgtt ttgctgtctg tatgcacaag tcttaacccc aaacaatttt
       E  L  A   R  S  S  Q
1601  tcagtatctc atggaacttg acaaacgacc acattattta tctaatggca tatcatccat tttgtagcat ttttagtctt gctgattatg ttatctttct
1701  tgtaaatgca gTTTATTGCT TCGTTCTACC AACACGTAGT GGTCCAAAGA CGGgtacaac tctttcaaac aagtcctttt tctgtgctgc atcttatact
                   F  I  A   S  F  Y   Q  H  V   V  V  Q  R   R
                SALK_016767 (om47-1)
1801  agtcttccat atataaagtt tagcacatct ttggctcaat atgaaatcga tttaatggac ttggaacagG TGCAAAATCC TTTCGAAGAA AACCAAGTAG
                   1820bp                                                      V  Q  N   P  F  E  E   N  Q  V
1901  TTGGAATCAC AAACTACATT GATTTTGGTT TTGAGCTACA ATCGAGgtag tgaacatctc taagtaaaaa aaaaaaaaac attgtagcta ctgtttgtct
       V  G  I   T  N  Y  I   D  F  G   F  E  L   Q  S
2001  atacagaaac agaactttat atttcgtctt aatattgaag GGTTGATGAT TCCAAGACGC CACCCAATGC CCCAGATTCT TTATTGCAGG TGGCTGCGTC
                                                R  V  D  D   S  K  T   P  P  N   A  P  D  S   L  L  Q   V  A  A
2101  TTGGCAGGCC AACAAGAACT TTTTGCTGAA Ggtataaaaa aatatttacc ttatcgagtt actcaaaaag aaaccattta tctcaaattt tgctcagtat
      S  W  Q  A   N  K  N   F  L  L   K
2201  gtaacatttt caacttttgc agGGTAAAGT CGGAGCTCAT AGCTCAACAT TGTCATTAGC ATTCAAGTCG TGGTGGAAAC CGTCTTTCGC ATTCAATATT
                               G  K   V  G  A  H   S  S  T   L  S  L   A  F  K  S   W  W  K   P  S  F   A  F  N  I
                                                                     GABI_369G03 (om47-2)
2301  TCAGgtatgc gttgataaat aacctgaatg cagcgaaatc aagatgaaca taggttcttg gtatttgaac atgtttggac tcattttta tgcagCAACA
       S                                                         2357bp                                    A  T
2401  ACTAATCATA GGACTGGAAA TGTCCAATGT GGGTTCGGTC TACGTGTTGA TAACTTAAGA GAAGCCAGgt cagtccattt gtgacaagtt taaaattaaa
       T  N  H   R  T  G   N  V  Q  C   G  F  G   L  R  V   D  N  L  R   E  A
2501  tcaaccctct ttagcttcat gatcttcttc acattgtttt attttgtgaa attgcagTTA CCAAAGAGCT GATCCAAACT TTGTAATGCT GACACCGAAC
                                                                 S   Y  Q  R  A   D  P  N   F  V  M   L  T  P  N
2601  AAAGAACATT TAGCTGAAGG GATTGTGTGG AAAATGGGGA AGAGACCAAT GTATCAAGCC GATGTGGACG CAGAGAATTT TAGTGAGCTG CCAAAAGAAC
       K  E  H   L  A  E   G  I  V  W   K  M  G   K  R  P   M  Y  Q  A   D  V  D   A  E  N   F  S  E  L   P  K  E
2701  TTAGACCGTC CCAGAAGATT CTCTAAacta caccattaat ctttttgcac ttttatctag tagatttaga tatcaaccaa gtcaagtatt taataagttc
       L  R  P   S  Q  K  I   L
      gacgaaagaa gaattactta ggaataacca tttttactct tatggagtac aagtgtttag tgttcaaaat taggagtgga ttatctggtt taaactctta
      agtttggtct ttttgggctt ttgatttttc gaagttctta aatgaagcga aatcctgatt tgtttttgga cttttcattc ggtttggaac gatatttata
      tgggatgata tattacgggt ---------- ---------- ---------- ---------- ---------- ---------- ---------- ----------
```

Supplemental Figure 1. T-DNA insertion position as determined by sequencing for the T-DNA lines used in this study.

The genomic DNA sequence, the intron and exon sequences and predicted protein sequence are shown. The position of the T-DNA inserts in lines SALK_016767 (om47-1) and GABI_369G03 (om47-2) are shown at 1820 bp and 2357 bp, respectively, with the base A of the ATG start site designated bp1. Sequence in black: UTR (untranslated region), red uppercase: exon and blue lowercase: intron. The protein sequence is presented in green below the nucleotide sequence. The start and stop codons are indicted by a blue box. The arrows indicate the exact T-DNA insertion site in SALK_016767 (om47-1) and GABI_369G03 (om47-2).

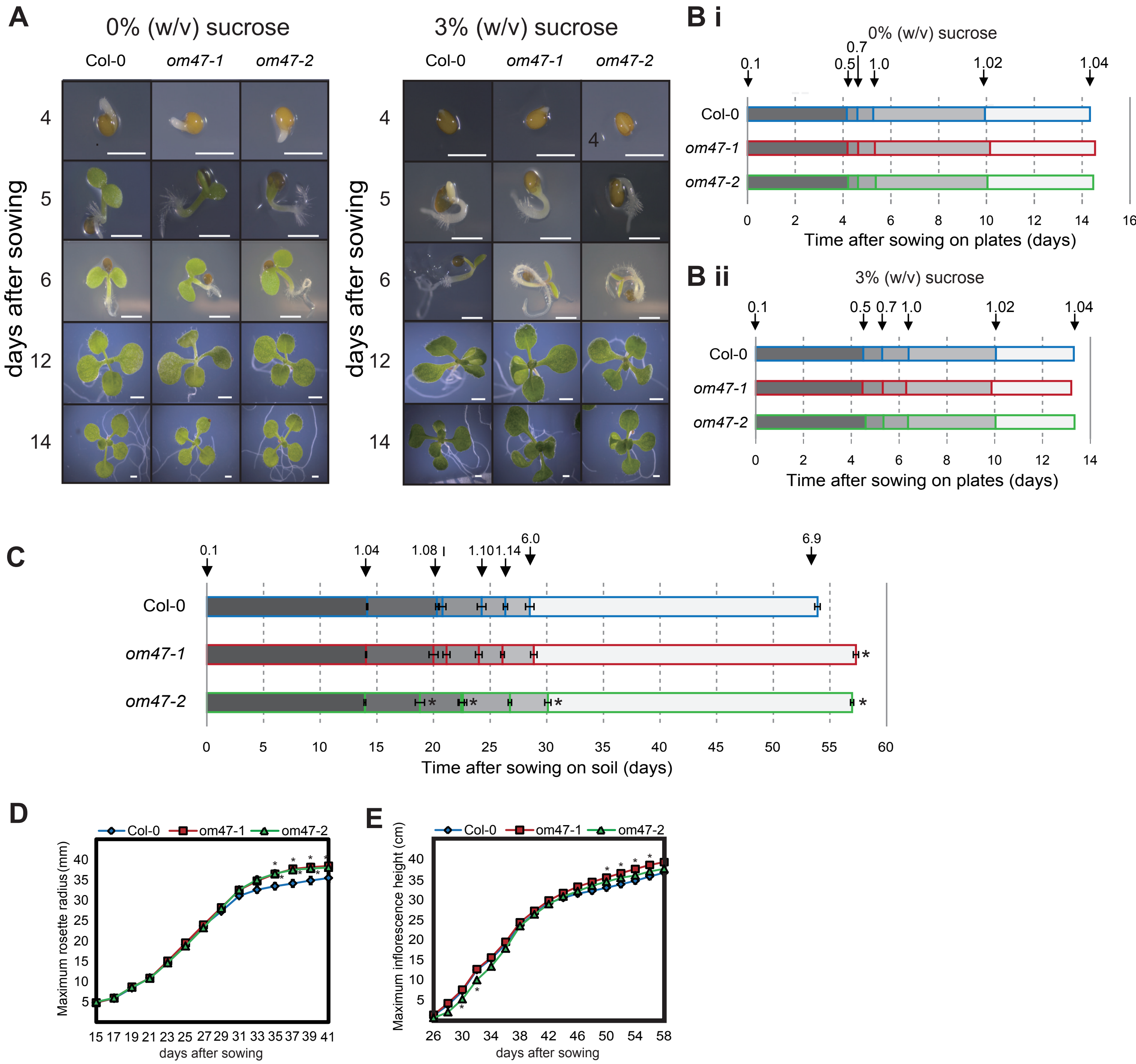

Supplemental Figure 2. Plate-based development stages of Arabidopsis thaliana om47 mutants.

A. Representative seedling images of Arabidopsis thaliana wild-type (Columbia-0, Col-0) and om47 mutants on plates containing no sucrose (0% sucrose) and 3% sucrose over 14 days post-germination. Scale bars indicate 1mm. Days are the period after sowing including a 3-d stratification at 4°C.

B. Development stage analysis of Col-0, om47-1 and om47-2 grown on MS media with 3% (w/v) sucrose (i) or without sucrose (ii) for 14 days. Arrows define the time (days after sowing) that Col-0 plants have reached the growth stages as defined by Boyes et al. (2001). Boxes indicate the time between the growth stages, and shading indicates the period of each growth stage. Stage 0.1, imbibition; stage 0.5, radical emergence; stage 0.7, hypocotyl emerge from seed coat; stage 1.0, cotyledons fully opened; stage 1.02, two rosette leaves >1 mm in length; stage 1.04, four rosette leaves >1mm in length. Data are given as averages ±SE for 50 plants. Days are the period after sowing including a 3-d stratification at 4°C.

C. Soil based growth stage progression for Arabidopsis thaliana wild type (Col-0) and om47 mutants over their entire life cycle as defined by Boyes et al. (2001). Arrows define the time points (days after sowing) when wild-type plants reached the growth stages indicated. Boxes represent the time elapsed (days) between the occurrences of successive growth stages. Junctions between boxes of different shading indicate the occurrence of a growth stage (n = 20, data are given as averages, error bars represent ±SE, asterisks identify significant differences ($P \leq 0.05$) compared to wild type at each growth stage as determined by Student's t-test). Days are the period after sowing including a 2-d stratification at 4°C.

D. Maximum rosette radius (upper panel) and inflorescence height (lower panel) of Arabidopsis thaliana wild-type (Columbia-0, Col-0, blue diamonds), om47-1 (red squares) and om47-2 (green triangles) across an entire life cycle (n = 20, data are given as averages, error bars represent ±SE, *; significant differences ($P \leq 0.05$) compared to wild-type at each growth stage as determined by Student's t-test). Days are the period after sowing after a 2-d stratification at 4°C.

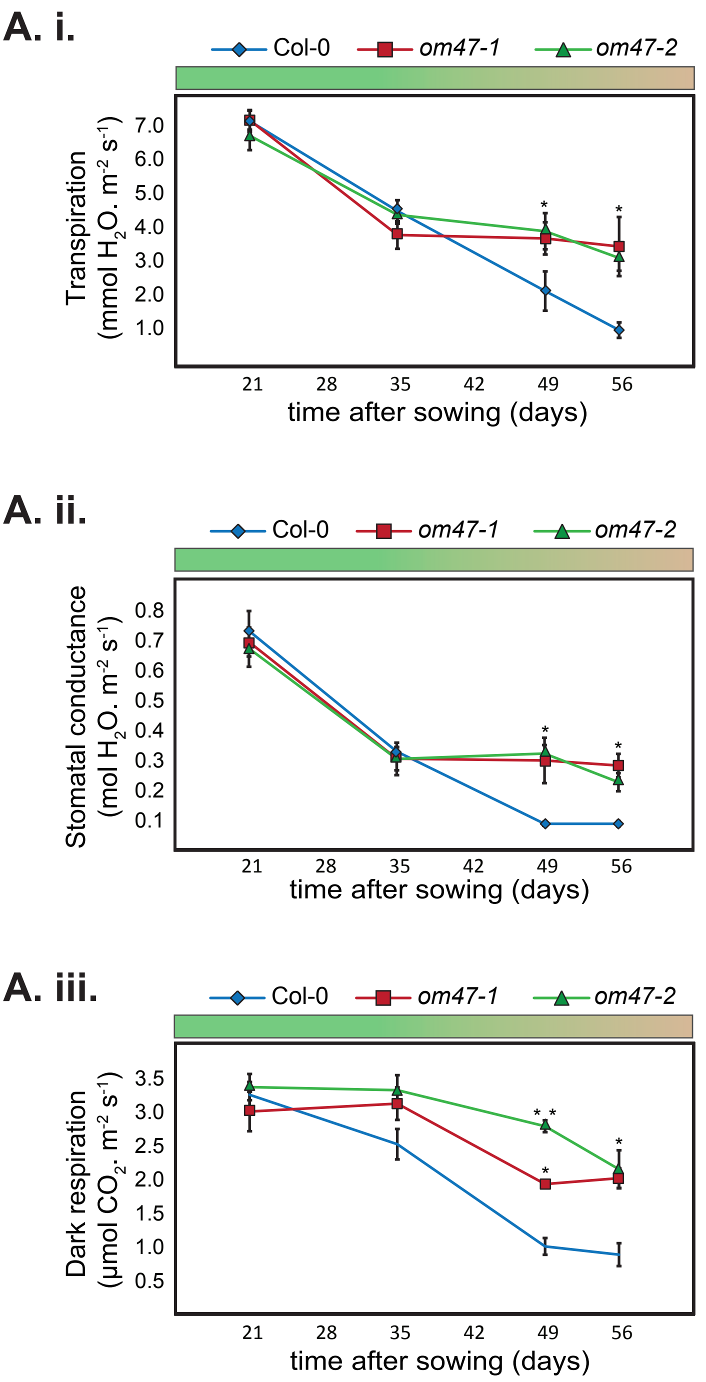

Supplemental Figure 3. Arabidopsis thaliana om47 mutants have enhanced gas exchange parameters during senescence.
(i.) Transpiration, **(ii.)** stomatal conductance and **(iii.)** dark respiration of Arabidopsis thaliana wild type (Columbia-0) and om47 mutants over 56 days under control (16/8 h photoperiod) conditions. Coloured bar represents stage of senescence through-out experiment (n = 4, data are given as averages, error bars represent ±SE, *; significant differences ($P \leq 0.05$) compared to wild-type as determined by Student's t-test).

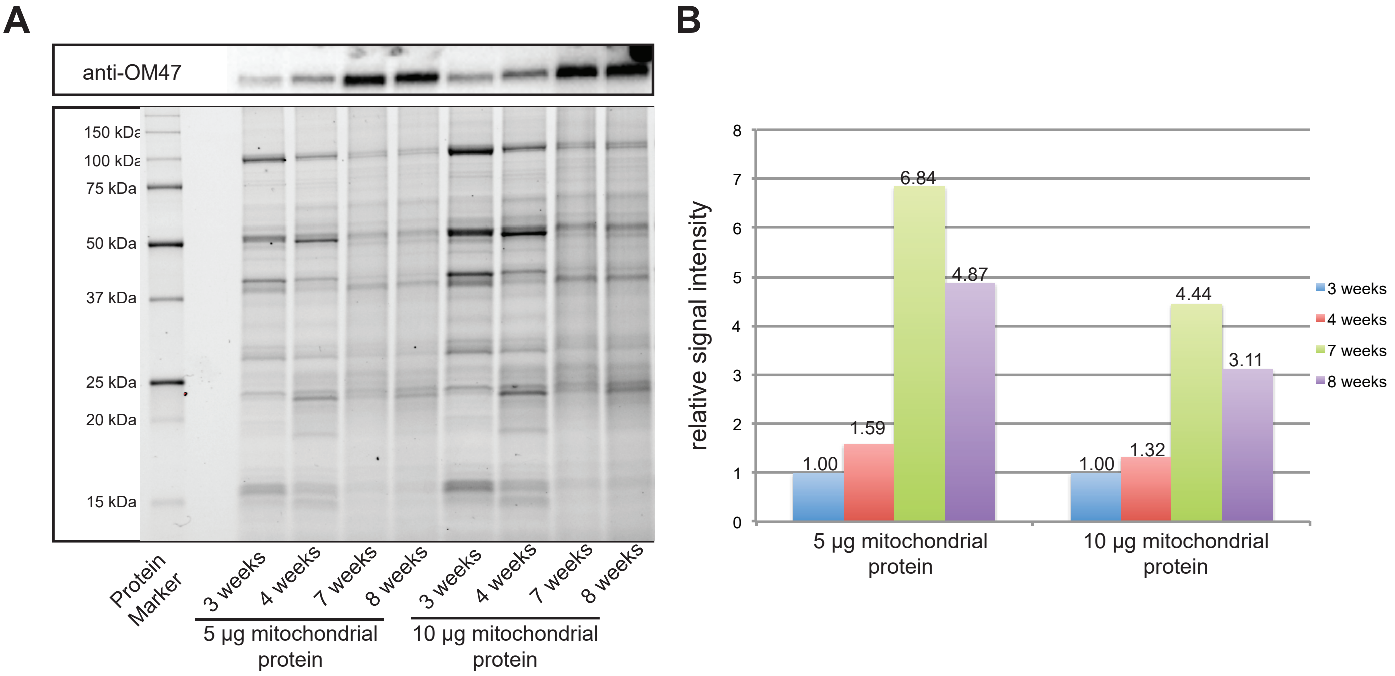

Supplemental Figure 4: Relative protein abundance of OM47 in mitochondria isolated from *om47-2* mutant plants at different developmental stages

The abundance of OM47 increased by at least 4-fold in 7-weeks old and 3-fold in 8-weeks old plants compared to the 3-weeks old plants, while the abundance only increase by 1.5-fold at 4-weeks. These results correlated with results for the transcript abundance (Figure 8), together indicating a compensatory increase in OM47 expression at later developmental stages on the transcript and protein level.

A) Immunoblot (upper panel) showing detection of OM47 from mitochondria of the *om47-2* mutant isolated at the indicated plant ages. The lower panel shows the corresponding SDS-PAGE gel as loading control, also revealing the varying protein composition in mitochondria at different developmental stages. Amounts of mitochondrial protein loaded (5 µg and 10 µg) and apparent molecular masses of molecular weight standard are indicated.

B) Quantification of signal intensities of the immunoblot shown in A. Indicated are fold changes relative to the 3-weeks old plants.

Supplemental Table 1.1. Digital PCR primers used to amplify transcripts.

Locus ID	Gene name	Primer name	Sequence (5'- 3')
AT3G44880	PAO	PAO_RT_FW	ACTGTCTTAACCAAGCGTCAGATG
		PAO_RT_REV	GCGCCAACGAGAAACTTCTTGAG
AT4G22920	SGR1	SGR1_RT_REV	ATCCTTCAACGCTCCCTAGGAC
		SGR1_RT_FW	TTGCCCATCCTTGCAACTGAG
AT5G45890	SAG12	SAG12_RT_REV	TCCTTACAAAGGCGAAGACGCTAC
		SAG12_RT_FW	ACCGGGACATCCTCATAACCTG
AT5G38410	RBCS3B	RBCS3B_RT_FW	ACCTTAGTGACGTCGAATTGGC
		RBCS3B_RT_REV	CGGTACACAAATCCGTGCTCTAAC
AT3G27930	OM47	AtOM47_RT_FW	ATGGGGTTGAGATATGGCTCAACG
		AtOM47_RT_REV	ATCTTGCTTACAAGCCATGCATGC
AT5G25760	UBC21	AtUBC_RT_FW	CTGCGACTCAGGGAATCTTCTA
		AtUBC_RT_REV	TTGTGCCATTGAATTGAACCC

Supplemental Table 1.2.PCR primers used to identify the T-DNA insertion alleles.

Gene name	T-DNA insertion line	Primer name	Sequence (5' - 3')
		SALK_LB1.3 (BP primer for *om47-1*)	ATTTTGCCGATTTCGGAAC
		GABI (BP primer for *om47-2*)	CCCATTTGGACGTGAATGTAGACAC
OM47	SALK_016767 (*om47-1*)	SALK_016767 LP	GTCACAAATGGACTGACCTGG
		SALK_016767 RP	TTTGGCAAAGTACACAGACCC
OM47	GABI_369G03 (*om47-2*)	GABI_369G03 LP	CAAGTCCTTTTTCTGTGCTGC
		GABI_369G03 RP	TTTAGAGAATCTTCTGGGACGG

Supplemental Table 2. List of all antibodies used

Antibody Name	AT number	Source	Reference/Order number	Specificity
OM47	At3g27930	Jim Whelan	this manuscript	detect the protein encoded by AT3g27930
ELM1	AT5G22350	Jim Whelan	this manuscript	detect the protein encoded by AT2G22350
NDB2	AT4g05020	Kathleen Soole	Soole and Smith., 2015	detect the protein encoded by AT4g05020
Ndufs4	At5g67590	Etienne Meyer	Meyer et al., 2009	detect the protein encoded by At5g67590
KDSB	AT1G53000	Jim Whelan	Duncan O et al., 2011	detect the protein encoded by AT1g53000
a-subunit of ATP synthase	At5g08670	Agrisera	AS05 085	detect the protein encoded by At5g08670
COXII	AtMG00160	Agrisera	AS04 0543A	detect the protein encoded by AtMG00160
RISP	At5g13430	Jim Whelan	Carrie C et al., 2010	detect the protein encoded by At5g13430
AOX	At3g22370	Tom Elthon	Elthon et al., 1989	detect the protein encoded by At3g22370
Cyt c	At1g22840	Agrisera	AS08 343A	detect the protein encoded by At1g22840
SAM50	At3g11070	Jim Whelan	Carrie C et al., 2010	detect the protein encoded by At3g11070
Porin	At3g01280	Tom Elthon	Elthon et al., 1989	detect the protein encoded by At3g01280
Tom40	At3g20000.1	Jim Whelan	Carrie C et al., 2010	detect the protein encoded by At3g20000.1
Tim50	AT1G55900	Jim Whelan	Wang Y et al., 2012	detect the protein encoded by AT1G55900
Erv1	At1g49880	Jim Whelan	Carrie C et al., 2010	detect the protein encoded by At1g49880
Tim9	At3g46560	Jim Whelan	Wang Y et al., 2012	detect the protein encoded by At3g46560
